# Structural insights into the functions of the FANCM-FAAP24 complex in DNA repair

**DOI:** 10.1093/nar/gkt788

**Published:** 2013-09-03

**Authors:** Hui Yang, Tianlong Zhang, Ye Tao, Fang Wang, Liang Tong, Jianping Ding

**Affiliations:** ^1^State Key Laboratory of Molecular Biology, Institute of Biochemistry and Cell Biology, Shanghai Institutes for Biological Sciences, Chinese Academy of Sciences, 320 Yue-Yang Road, Shanghai 200031, China, ^2^Graduate School of Chinese Academy of Sciences, 320 Yue-Yang Road, Shanghai 200031, China and ^3^Department of Biological Sciences, Columbia University, New York, NY 10027, USA

## Abstract

Fanconi anemia (FA) is a genetically heterogeneous disorder associated with deficiencies in the FA complementation group network. FA complementation group M (FANCM) and FA-associated protein 24 kDa (FAAP24) form a stable complex to anchor the FA core complex to chromatin in repairing DNA interstrand crosslinks. Here, we report the first crystal structure of the C-terminal segment of FANCM in complex with FAAP24. The C-terminal segment of FANCM and FAAP24 both consist of a nuclease domain at the N-terminus and a tandem helix-hairpin-helix (HhH)_2_ domain at the C-terminus. The FANCM-FAAP24 complex exhibits a similar architecture as that of ApXPF. However, the variations of several key residues and the electrostatic property at the active-site region render a catalytically inactive nuclease domain of FANCM, accounting for the lack of nuclease activity. We also show that the first HhH motif of FAAP24 is a potential binding site for DNA, which plays a critical role in targeting FANCM-FAAP24 to chromatin. These results reveal the mechanistic insights into the functions of FANCM-FAAP24 in DNA repair.

## INTRODUCTION

Fanconi anemia (FA) is a rare genetic disease characterized by bone marrow failure, developmental defects and increased incidence of cancers (1,2). FA is genetically heterogeneous and can be caused by defects in any of 15 FA genes. The FA proteins function in resolving DNA interstrand crosslinks (ICLs) and stalled replication forks during replication via the FA pathway (1,2). As the central event of the FA pathway, the eight upstream FA proteins (FANCA, FANCB, FANCC, FANCE, FANCF, FANCG, FANCL and FANCM) cooperate with five FA-associated proteins (FAAP20, FAAP24, FAAP100, MHF1 and MHF2) to form the FA core complex, which acts as an E3 ubiquitin ligase to monoubiquitinate the FANCI-FANCD2 complex and then activates the downstream DNA repair pathways (3–14).

FANCM is a key component of the FA core complex, which contains a DEAH helicase domain at the N-terminus and a putative nuclease domain and a tandem helix-hairpin-helix (HhH)_2_ motif domain at the C-terminus. The DEAH helicase domain harbors an ATP-dependent DNA-remodeling translocase activity, which is important for the FA core complex to monoubiquitinate FANCI-FANCD2 (15–19). FANCM can interact with the FANCM-associated histone-fold proteins 1 and 2 (MHF1-MHF2) complex via the region following the helicase domain (residues 661–800) and with FAAP24 via the C-terminal domains to form two stable complexes to bind different DNA substrates, which are crucial for FANCM to facilitate repair of ICLs (3–5). FANCM can also interact with the RMI1-RMI2 complex via its MM2 motif (residues 1218–1251) to recruit the BLM-RMI1-Topo IIIα dissolvasome to ICL-stalled replication forks (20–22).

The FANCM-FAAP24 complex binds preferentially to single-stranded DNA (ssDNA). It plays a key role in the recruitment of the FA core complex to damaged DNA and the monoubiquitylation of FANCD2 (5,18). It is also essential for ataxia-telangiectasia and Rad-3-related (ATR)-mediated S phase checkpoint signaling (23–25). Moreover, in addition to their coordinated functions, the two proteins have non-overlapping and distinct functions: FAAP24 promotes ATR-mediated checkpoint activation particular in response to DNA ICL agents, whereas FANCM participates in recombination-independent ICL repair by facilitating recruitment of lesion incision activities (26). The FANCM-MHF complex binds preferentially to branched DNA and double-stranded DNA (dsDNA) *in vitro* and stimulates the DNA branch migration and replication fork reversal activities of FANCM (3,4). The MHF1-MHF2 complex assumes a heterotetrameric architecture similar to that of the histone (H3-H4)_2_ heterotetramer and interacts with FANCM to form a binding site for DNA (27,28).

FANCM and FAAP24 are suggested to belong to the XPF family (5,16). All members of this family contain an excision repair cross-complementation group 4 (ERCC4) nuclease domain and a tandem helix-hairpin-helix (HhH)_2_ motif domain and exist as heterodimers in eukaryotes and homodimers in archaea. These proteins can be divided into two groups: catalytic members and non-catalytic members. The catalytic members such as XPF and Mus81 contain a conserved GDX_n_ERKX_3_D motif at the active site of the nuclease domain and exhibit nuclease activity, and the non-catalytic members such as Eme1, Eme2 and ERCC1 lack the catalytic motif (29,30). Previous data showed that FANCM has a divergent sequence CDX_n_ERRX_3_E at the equivalent region of the catalytic motif and FAAP24 lacks the catalytic motif, and the FANCM-FAAP24 complex has no detectable nuclease activity *in vitro* (5,16). The (HhH)_2_ domain is composed of two tandem HhH motifs, which can mediate the binding of different DNA structures and dimerization of the two proteins (31–34).

We report here the crystal structure of human FANCM-FAAP24 complex, which together with the biological data provide the molecular insights into its functions in DNA repair. The FANCM-FAAP24 heterodimer structurally resembles the ApXPF homodimer. Although the putative active site of FANCM has a similar structure as that of XPF, differences of several key residues and the electrostatic surface of the surrounding region render an inactive nuclease domain, accounting for the lack of nuclease activity. The first HhH motif of FAAP24 is a potential DNA binding site, which is essential for proper localization of FANCM-FAAP24 to chromatin in DNA repair.

## MATERIALS AND METHODS

### Cloning, expression and purification

The gene fragments encoding the C-terminal regions of human FANCM (residues 1727–2048, 1799–2048 and 1813–2031) and human FAAP24 (full-length and residues 17–215) were inserted into the pET-Duet plasmid (Novagen) and the pET-28 a plasmid (Novagen), respectively; the latter attaches a His_6_ tag at the C-terminal of FAAP24. The two plasmids were co-transformed into *Escherichia coli* BL21 (DE3) Codon-Plus strain (Novagen). The transformed cells were grown at 37°C in lysogeny broth (LB) medium until OD_600_ reached 0.8 and then induced with 0.2 mM isopropyl β-D-1-thiogalactopyranoside. The cells were collected by centrifugation, suspended in buffer A [20 mM Tris–HCl (pH 8.0), 500 mM NaCl, 10% glycerol and 1 mM phenylmethylsulfonyl fluoride] and lysed by sonication. The FANCM-FAAP24 complex was purified by affinity chromatography using a Ni-NTA column (Qiagen) with buffer A supplemented with 20 mM imidazole and 200 mM imidazole serving as washing buffer and elution buffer, respectively. The elution sample was further purified by gel filtration using a Superdex 200 16/60 column (GE Healthcare) pre-equilibrated with buffer B [10 mM HEPES (pH 7.5), 100 mM NaCl and 2 mM MgCl_2_]. For the Se-Met derivative proteins, the cells were grown in M9 medium supplemented with amino acids Lys, Thr, Phe, Leu, Ile, Val, Se-Met and 1% lactose. The FANCM and FAAP24 mutants containing different point mutations were generated using the QuikChange® Site-Directed Mutagenesis Kit (Strategene). The resultant protein samples were of >95% purity as analyzed by SDS–PAGE.

### Crystallization, data collection and structure determination

Crystallization was performed using the hanging drop vapor diffusion method at 20°C. Crystals of both native and Se-Met FANCM-FAAP24 were grown from drops consisting of 1 µl of protein solution (∼6 mg/ml) and 1 µl of reservoir solution containing 1.0 M (NH_4_)_2_SO_4_ and 0.1 M HEPES (pH 7.2). For diffraction data collection, the crystals were cryoprotected using the reservoir solution supplemented with 30% glycol and then flash-cooled into liquid nitrogen. Three Se-derivative datasets of 3.4 Å resolution and a native data set of 2.9 Å resolution were collected at 100 K at beamline 17 U of Shanghai Synchrotron Radiation Facility, China. The diffraction data were processed with HKL2000 (35). The statistics of the diffraction data are summarized in Table 1.

**Table 1.** Summary of diffraction data and structure refinement statistics

	Peak	Inflection	Remote	Native
Diffraction data				
Wavelength (Å)	0.9795	0.9792	0.9568	0.9796
Space group	*I*222	*I*222	*I*222	*I*222
Cell parameters				
*a* (Å)	80.1	80.3	80.0	79.2
*b* (Å)	98.2	98.2	98.1	98.1
*c* (Å)	139.1	139.1	139.0	140.2
Resolution (Å)	50.0–3.40	50.0–3.40	50.0–3.40	50.0–2.90
	(3.52–3.40)^a^	(3.52–3.40)	(3.52–3.40)	(3.00–2.90)
Observed reflections	38 469	38 197	38 209	40 533
Unique reflections (I/σ(I) > 0)	7996	7965	7942	12 155
Average redundancy	4.8 (4.9)	4.8 (4.8)	4.8 (4.9)	3.3 (3.4)
Average I/σ(I)	14.4 (3.4)	13.8 (2.7)	14.4 (4.3)	20.5 (2.8)
Completeness (%)	99.8 (100.0)	99.8 (100.0)	99.7 (99.9)	97.6 (97.5)
*R*_merge_ (%)^b^	15.1 (67.3)	15.6 (85.6)	13.8 (53.4)	6.8 (50.2)
Refinement and structure model				
Reflections (*Fo≥0σ(Fo*))				
Working set				11 554
Test set				598
*R* factor/Free *R* factor (%)^c^				25.4/29.9
No. of Protein atoms				3,058
No. of Water atoms				8
Average B factor (Å^2^)				
All atoms				69.7
FANCM main chain/side chain				62.2/64.6
FAAP24 main chain/side chain				75.7/77.2
Water				42.6
RMS deviations				
Bond lengths (Å)				0.006
Bond angles (°)				1.1
Ramachandran plot (%)				
Most favoured				90.5
Allowed				8.4
Generously allowed				1.1

^a^Numbers in parentheses represent the highest resolution shell.

^b^Rmerge=∑_hkl_∑_i_|I_i_(hkl)−<I(hkl)>|/∑_hkl_∑_i_I_i_(hkl).

^c^R=∑_hkl_||F_o_|−|F_c_||/∑_hkl_|F_o_|.

The structure of FANCM-FAAP24 was solved using the multi-wavelength anomalous dispersion method using Phenix (36), which identified 7 Se atoms and yielded a figure of merit of 0.44. The structure model was refined against the native data set using Phenix (36) and Refmac5 (37). Model building was performed using Coot (38). The final structure model contains residues 1815–2030 of FANCM and residues 18–213 of FAAP24, except that three surface exposed loops (residues 1903–1915 and 1965–1969 of FANCM, and residues 147–152 of FAAP24) could not be modeled owing to poor electron density. Structure analysis was carried out using programs in CCP4 (39) and the PISA server (40). All the structure figures were prepared using Pymol (http://www.pymol.org). The statistics of the structure refinement and the quality of the final structure model are also summarized in Table 1.

### Electrophoretic mobility shift assay

Electrophoretic mobility shift assays (EMSA) were performed as described previously (5). The ssDNA, dsDNA and splayed-arm DNA substrates were also prepared as described previously (5,41). For comparison, all DNAs were labeled with 5′-biotin in oligo 1 and oligo 3. Reaction mixture (20 µl) contained 10 nM 5′-biotin-labeled DNA in a buffer containing 25 mM Tris–HCl (pH 7.5), 5 mM EDTA and 6% glycerol. Reactions were initiated by adding different amounts of FANCM-FAAP24 and then incubated for 30 min at 4°C. Reaction mixture was loaded on a 6% neutral polyacrylamide gel prepared in 0.5 x TBE buffer (45 mM Tris–borate (pH 8.0) and 1 mM EDTA). Gels were run in 0.5x TBE buffer at 100 V for 100–120 min at 4°C and then transferred to a positively charged nylon membrane (GE Healthcare) in 0.5x TEB buffer at 100 V for 60 min at 4°C. The nylon membrane was cross-linked at 120 mJ/cm^2^ using UVC 5000 Crosslinker (Hofer). The biotin-labeled DNA was detected by LightShift Chemiluminescent EMSA Kit (Thermo Scientific).

### Cell culture, transfection and immunofluorescence

The genes of the full-length FANCM and the C-terminal segment of FANCM (residues 1799–2048, FANCM_L2_) in wild-type, truncated and mutant forms were subcloned into the pEGFP-C3 vector (Clontech), which attaches an enhanced green fluorescent protein (EGFP) tag at the N-terminus, and the genes of the full-length FAAP24 in wild-type, truncated and mutant forms into the pcDNA3 vector (Invitrogen), which attaches a Flag tag at the N-terminus. HEK 293 T cells were cultured on coverslips in Dulbecco’s modified Eagle’s medium (Hyclone) supplemented with 10% fetal bovine serum (Biochrom AG) and transfected with 2 µg of plasmids using Lipofectamine 2000 (Invitrogen). Twenty-four hours after transfection, the cells were fixed with 4% paraformaldehyde (Sigma), washed three times with phosphate buffered saline (PBS) and permeabilized using PBS supplemented with 0.1% Triton X-100 and then blocked using 2% bovine serum albumin in PBS supplemented with 0.1% Triton X-100. Flag-FAAP24 was stained with mouse anti-Flag M2 mAb (1:1000, Sigma) followed by Alexa Fluor 647 goat anti-mouse mAb (1:1000, Invitrogen). DNA was stained with 4′,6-diamidino-2-phenylindole (DAPI, 1:2000, Sigma). Images were acquired utilizing a 63X oil immersion lens on a Leica TCS SP5 II confocal microscope (Leica).

## RESULTS AND DISCUSSION

### Structure of the FANCM-FAAP24 complex

**Figure 1.**
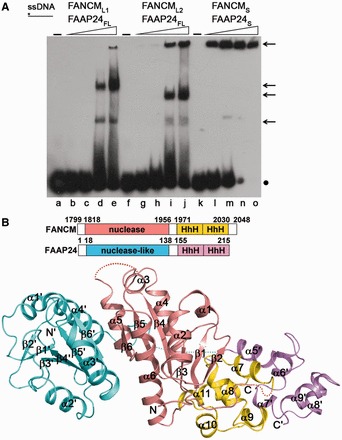
Structure of the FANCM-FAAP24 complex. (**A**) EMSA of the DNA-binding ability of FANCM-FAAP24 on ssDNA. The reaction contained the biotin-labeled ssDNA (10 nM) without or with increasing amounts of FANCM-FAAP24 (0.0 µM, lanes a, f and k; 0.05 µM, lanes b, g and l; 0.1 µM, lanes c, h and m; 0.4 µM, lanes d, i and n; and 0.8 µM, lanes e, j and o). The asterisk represents the biotin label at the DNA 5′ end. The arrows indicate the shifted bands of the protein–DNA complexes, and the dot represents the free DNA probe. (**B**) Ribbon representation of the overall structure of FANCM-FAAP24. The nuclease and the (HhH)_2_ domains of FANCM are colored in salmon and yellow, and those of FAAP24 in cyan and violet, respectively. The secondary structure elements are labeled with those of FAAP24 designated with apostrophes. The disordered regions are indicated with dotted lines.

It was reported previously that two forms of the C-terminal fragment of human FANCM (residues 1727–2048, FANCM_L1_; residues 1799–2048, FANCM_L2_) can form stable complexes with full-length FAAP24 (residues 1–215, FAAP24_FL_), which exhibit binding activities to different types of DNA structures with preferences for ssDNA, splayed-arm DNA and a 3′-flap DNA 5–10-fold better than for dsDNA (5). We obtained these two forms of the FANCM-FAAP24 complex with high purity, stability and homogeneity and confirmed their binding to different types of DNA structures (Figure 1A and Supplementary Figure S1), but failed to obtain any crystals. We then constructed a slightly shorter form of FANCM (residues 1813–2031, FANCM_S_) and an N-terminal truncated form of FAAP24 (residues 17–215, FAAP24_S_), and the resultant FANCM_S_-FAAP24_S_ complex led to successful crystallization and structure determination of the complex (Figure 1B). We also confirm that the FANCM_S_-FAAP24_S_ complex maintains the capability to bind different types of DNA substrates (Figure 1B and Supplementary Figure S1). It is noteworthy that there are several shifted bands in the EMSA results. As the FANCM-FAAP24 complex can bind different types of DNA structures in a non-sequence-specific way, and the DNA substrates are relatively long, it is possible that a single DNA substrate might bind one or more protein molecules under the assay condition, leading to multiple shifted bands. This speculation is supported in part by the observation that the bands with larger molecular masses appear to be more intense at higher protein concentration. The FANCM_S_-FAAP24_S_ complex appears to bind the DNA substrates to form large protein–DNA complexes with higher molecular masses, which barely move in the gels. For simplicity, we will refer to this complex as the FANCM-FAAP24 complex hereafter unless otherwise specified.

The crystal structure of the FANCM-FAAP24 complex was solved using the multi-wavelength anomalous dispersion method and refined to 2.9 Å resolution (Figure 1B and Table 1). There is one FANCM-FAAP24 complex in the asymmetric unit, which contains residues 1815–2030 of FANCM and residues 18–213 of FAAP24 except that three solvent-exposed loops (residues 1903–1915 and 1965–1969 of FANCM and residues 147–152 of FAAP24) are disordered. The N-terminal ends of both FANCM and FAAP24 are not located in the protein–protein interface (Figure 1B), consistent with our biochemical data showing that the N-terminal truncations of these proteins do not affect the stability of the complex and the binding to DNA (Figure 1A and Supplementary Figure S1).

Both FANCM and FAAP24 are composed of a nuclease domain at the N-terminus and an (HhH)_2_ domain at the C-terminus, although they share only 20% sequence identity. The nuclease domains of both FANCM and FAAP24 contain a central six-stranded β-sheet flanked by helices on both sides (Figure 1B). The (HhH)_2_ domains of both FANCM and FAAP24 are composed of five helices, namely, α7 -α11 and α5′-α9′, respectively (components of FAAP24 are designated by apostrophes) (Figure 1B). The overall structures of the equivalent domains in the two proteins are similar with root-mean-square deviation of 1.9 Å and 1.6 Å, respectively (Figure 1B and Supplementary Figure S2A and B). However, the relative position and orientation of the two domains are significantly different: the nuclease domain of FANCM is sandwiched between and interacts with the (HhH)_2_ domain of FANCM and the nuclease domain of FAAP24, whereas the nuclease domain of FAAP24 is far apart from and has no interaction with the (HhH)_2_ domain of FAAP24 (Figure 1B and Supplementary Figure S2C). The two nuclease domains and the two (HhH)_2_ domains form two compact heterodimers, respectively.

### Interactions between FANCM and FAAP24

**Figure 2.**
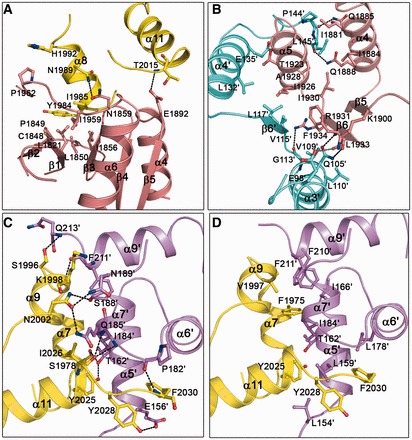
Interactions between FANCM and FAAP24. (**A**) Interactions between the nuclease and the (HhH)_2_ domains of FANCM. The interface is mainly mediated by residues from α4 and the β2-β3, β3-β4, α4-β5 and α6-α7 loops of the nuclease domain, and α8 and the α7-α8 and α10-α11 loops of the (HhH)_2_ domain. (**B**) Interactions between the nuclease domains of FANCM and FAAP24. The interface is mainly mediated by residues from α4, α5 and β6 of FANCM and α3′, α4′, β6′ and the α4′-α5′ loop of FAAP24. (**C**) Hydrophilic interactions and (**D**) hydrophobic interactions between the (HhH)_2_ domains of FANCM and FAAP24. The interface is mainly mediated by residues from α7, α9 and α11 of FANCM and α5′, α6′, α7′ and α9′ of FAAP24. The nuclease and the (HhH)_2_ domains of FANCM are colored in salmon and yellow, and those of FAAP24 in cyan and violet, respectively. The interacting residues are shown with ball-and-stick models. The hydrogen bonds are indicated with black dashed lines and the salt bridge with gray dashed line.

The interactions between the nuclease domain and the (HhH)_2_ domain of FANCM are mediated mainly by residues from α4 and the β2-β3, β3-β4, α4-β5 and α6-α7 loops of the nuclease domain, and α8 and the α7-α8 and α10-α11 loops of the (HhH)_2_ domain, which involve numerous hydrophobic contacts and two hydrogen bonds and bury a total of 655 Å^2^ solvent-accessible surface area (Figures 1B and 2A and Supplementary Table S1). The N-terminal of α8 of the (HhH)_2_ domain is embedded in a hydrophobic surface patch formed by the β2-β3, β3-β4, α6-α7 loops of the nuclease domain, which contribute more than half of the hydrophobic contacts and one hydrogen bond at this interface (Supplementary Table S1). Helix α4 and the following α4-β5 loop of the nuclease domain mainly interact with the α7-α8 and α10-α11 loops of the (HhH)_2_ domain, which also contribute nearly half of the hydrophobic contacts and one hydrogen bond at this interface (Supplementary Table S1).

The two nuclease domains form a heterodimer with pseudo 2-fold symmetry such that the C-terminal edges of the two β-sheets (β6) are in contact with each other (Figures 1B and 2B). The interaction interface involves mainly residues from α4, α5 and β6 of FANCM and α3′, α4′, β6′ and the α4′-α5′ loop of FAAP24 and buries a total of 1021 Å^2^ solvent-accessible surface area (Figures 1B and 2B and Supplementary Table S2). The interactions are largely hydrophobic but also comprise 5 hydrogen bonds and 1 salt bridge. Specifically, the interactions between β6 and α5 of FANCM and β6′, α3′ and α4′ of FAAP24 contribute more than two-thirds of the hydrophobic contacts and four hydrophilic interactions at this interface. The interactions between α4 of FANCM and the α4′-α5′ loop of FAAP24 contribute the rest of the hydrophobic contacts and two hydrogen-bonding interactions.

The two (HhH)_2_ domains also form a heterodimer with pseudo 2-fold symmetry, and the interaction interface involves mainly residues from α7, α9 and α11, and the connecting loops of FANCM and α5′, α6′, α7′ and α9′, and the connecting loops of FAAP24, and buries a total of 950 Å^2^ solvent-accessible surface area (Figures 1B and 2C and D and Supplementary Table S2). Similarly, the interactions are largely hydrophobic but also comprise 11 hydrogen bonds. Helix α9 and the preceding loop of FANCM mainly interact with α7′, α9′, the α7′-α8′ loop, and the C-terminal of FAAP24, which contribute one fourth of the hydrophobic contacts and five hydrogen bonds at this interface. Helix α11 and the C-terminal of FANCM mainly interact with α5′, α7′, and the α4′-α5′ and α6′-α7′ loops of FAAP24, which contribute more than half of the hydrophobic contacts and five hydrogen bonds. The extensive hydrophilic and hydrophobic interactions between the two (HhH)_2_ domains are in agreement with the observation that the (HhH)_2_ domains mediate the dimerization in other XPF family members (31,32,34).

### Structural comparisons with other XPF family members

Previously, the structures of ApXPF (*Aeropyrum pernix*, Ap) and DrMus81-HsEme1 (*Danio rerio*, Dr; *Homo sapiens*, Hs) containing both the nuclease and the (HhH)_2_ domains, the dimeric (HhH)_2_ domains of human XPF and human XPF-ERCC1, and the central domain of human ERCC1 have been determined (31–34,42,43). Structural comparisons show that the nuclease and the (HhH)_2_ domains of both FANCM and FAAP24 resemble those of ApXPF, DrMus81 and HsEme1, with root-mean-square deviations of 1.5–2.5 Å (Supplementary Figure S3A and B). The structure of the FANCM-FAAP24 heterodimer has the highest similarity to that of the ApXPF homodimer (Supplementary Figure S3C and D), even though they share ∼25% sequence identity (Supplementary Figure S4A). It was suggested previously that FANCM and XPF may have a common ancestor with a helicase domain at the N-terminal and a nuclease domain at the C-terminal (16). Although the structure of human XPF-ERCC1 containing both the nuclease and the (HhH)_2_ domains is unavailable yet, the structural similarities between FANCM-FAAP24 and ApXPF and between the dimeric (HhH)_2_ domains of FANCM-FAAP24 and human XPF-ERCC1 support this notion and further suggest that the overall structure of XPF-ERCC1 should be similar to that of FANCM-FAAP24.

Structural comparisons also show that the position of the dimeric (HhH)_2_ domains relative to the dimeric nuclease domains in FANCM-FAAP24 is different from those in ApXPF and DrMus81-HsEme1. When these complexes are superimposed based on the dimeric nuclease domains, the position of the (HhH)_2_ domains in FANCM-FAAP24 differs by a rotation of 60° from to that in the dsDNA-bound ApXPF, a rotation of 125° from to that in the apo ApXFP, and a rotation of 125° from to that in DrMus81-HsEme1 (Supplementary Figure S3E–G). The previous structural data have shown that when dsDNA binds to ApXFP, the (HhH)_2_ domains are rotated toward the nuclease domains, and the dsDNA is bound in the surface groove formed between the nuclease and the (HhH)_2_ domains (31). The variations in the relative arrangements of the nuclease and the (HhH)_2_ domains in these complexes might reflect their differed binding abilities and/or specificities for different DNA structures.

### Molecular basis for the lack of nuclease activity

**Figure 3.**
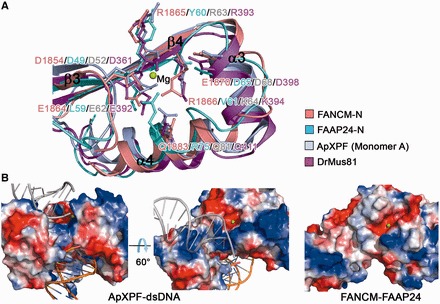
Structural comparison of the FANCM-FAAP24 complex with other XPF family members. (**A**) Comparison of the active sites of FANCM (salmon), FAAP24 (cyan), ApXPF (PDF code 2BGW, light blue) and DrMus81 (PDB code 2ZIU, magenta). Residues corresponding to the catalytic GDX_n_ERKX_3_D motif in FANCM, FAAP24, ApXPF and DrMus81 are shown with ball-and-stick models. Only the secondary structure elements of ApXPF are labeled. In the color coding scheme, the nuclease domains are abbreviated as N. Mg^2+^ in ApXPF-dsDNA is shown with a green sphere. (**B**) Electrostatic surface representations of the active sites in ApXPF-dsDNA and FANCM-FAAP24. The bound dsDNA in ApXPF-dsDNA is shown with orange ribbon and the modeled dsDNA with gray ribbon. ApXPF-dsDNA in the right panel and FANCM-FAAP24 are shown in the same orientation as these in Figure 3A. Mg^2+^ in FANCM-FAAP24 was modeled based on ApXPF-dsDNA and is shown with a green sphere.

The catalytic members of the XPF family contain a conserved GDX_n_ERKX_3_D motif in the active site of the nuclease domain, which is responsible for the nuclease activity. The previous mutagenesis studies of HsXPF showed that the first two acidic residues of the catalytic motif participate in metal binding; however, the functional roles of the two basic residues Arg and Lys and the last acidic residue Asp in catalysis have not been fully understood yet (29). Consistently, the structure of ApXPF-dsDNA showed that the side chains of the first two acidic residues (Asp52 and Glu62) and the main chain of the first basic residue (Arg63) are involved in the binding of Mg^2+^, but the side chains of the two basic residues (Arg63 and Lys64) and the last Asp (Asp68) are not (31). Sequence comparison of FAAP24 from different vertebrates shows that the catalytic motif is completely absent in FAAP24 (Supplementary Figure S4B). Although the nuclease domain of FAAP24 has a similar structure as that of ApXPF and DrMus81, the corresponding region of the catalytic motif becomes PDX_n_LYVX_3_D in FAAP24 (Figure 3A and Supplementary Figure S4A), suggesting the lack of capability to bind a metal ion that is required for the nuclease activity.

On the other hand, sequence comparison of FANCM from different vertebrates shows that the catalytic motif has evolved to CDX_n_ERRX_3_E in FANCM; in particular, the conserved Lys of the motif is substituted with Arg (Arg1866) and the last Asp is substituted with Glu (Glu1870) in human FANCM, both of which are shown to be involved in catalysis (Supplementary Figure S4A and C). Structural comparison shows that FANCM also has a similar active-site structure as that of ApXPF and DrMus81, and particularly all the key residues of the catalytic motif including the altered two residues assume similar side-chain conformations (Figure 3A). Nevertheless, no metal ion is found at the active site of FANCM, despite the fact that there is 2 mM MgCl_2_ in the protein buffer solution. We speculate that the altered residues in the catalytic motif of FANCM might change the coordination environment of the metal ion and/or the precise geometry of the active site, which could contribute in part to the lack of the nuclease activity of FANCM.

Furthermore, we found that the electrostatic surface surrounding the putative active site in FANCM-FAAP24 is substantially different from that in ApXPF and DrMus81-HsEme1 (Figure 3B and Supplementary Figure S5). The electrostatic surfaces surrounding the active site in ApXPF and DrMus81-HsEme1 are largely positively charged. In ApXFP-dsDNA, the dsDNA is bound in the surface groove between the nuclease and the (HhH)_2_ domains and interacts mainly with several basic residues and the G-I-G hairpin from the (HhH)_2_ domain of monomer A, but it is positioned distantly from the active site of the nuclease domain (Figure 3B and Supplementary Figure S3E). Structural analysis of ApXFP-dsDNA showed that there is a pseudo 2-fold symmetry between the two (HhH)_2_ domains, implying that the (HhH)_2_ domain of monomer B could also bind DNA via the equivalent region. The positively charged surface region formed by the equivalent basic residues of monomer B is located in the vicinity of the active site, and a modeled dsDNA could bind to this region and is positioned closely to the active site (Figure 3B) (31). In addition, structural analysis of the HsXPF (HhH)_2_-ssDNA complex showed that the basic residues in the equivalent regions of both (HhH)_2_ domains participate in the binding of ssDNA (Supplementary Figure S5) (31,33). Moreover, mutagenesis studies of DrMus81-HsEme1 suggested that several basic residues surrounding the active site are involved in DNA binding (Supplementary Figure S5) (32). These data together indicate that the positively charged surface surrounding the active site is involved in the binding of DNA, which is also essential for the nuclease activity.

In sharp contrast, structural analysis of FANCM-FAAP24 shows that the equivalent region surrounding the active site is relatively hydrophobic (Figure 3B). In addition, although the nuclease and the (HhH)_2_ domains form a surface groove on the top side and a surface cleft on the bottom side, both of them are also composed of largely acidic and hydrophobic residues. These results suggest that the active-site region in FANCM-FAAP24 is unable to bind a DNA substrate, which is in agreement with the previous biochemical data showing that the C-terminal segment of FANCM (FANCM_L1_) exhibits little DNA-binding ability to ssDNA, dsDNA or splayed-arm DNA (5). Furthermore, as all members of the XPF family in eukaryotes exist as heterodimers, we also examined the DNA-binding ability of the dimeric nuclease domains of FANCM-FAAP24. Our EMSA results show that the dimeric nuclease domains of FANCM-FAAP24 have no detectable DNA-binding ability to different types of DNA structures (Supplementary Figure S6), which is also consistent with the structural data. Taken together, we suggest that the variation of two key residues at the active site and the lack of DNA binding ability of the surrounding region render a catalytically inactive nuclease domain of FANCM and account for the lack of nuclease activity of FANCM-FAAP24.

As reported previously, FANCM-FAAP24 targets the FA core complex to stalled replication forks during DNA replication at S phase (5,18). For repairing stalled replication forks, Mus81-Eme1 is required for the first incision to form a double-strand break (DSB) (44), and XPF-ERCC1 is required for the second incision (45–47). Thus, it seems that the nuclease activity of FANCM-FAAP24 is not required in the fork cleavage reaction. It is possible that the nuclease domain of FANCM was degenerated and became catalytically inactive during evolution to avoid redundant function, and FANCM may function mainly via its translocase activity in DNA repair. Indeed, it was recently shown that in addition to its coordinated function with FAAP24 in the activation of the FA pathway, FANCM can function alone in recombination-independent ICL repair by facilitating recruitment of lesion incision activities via its translocase activity (26).

### Potential DNA binding site

**Figure 4.**
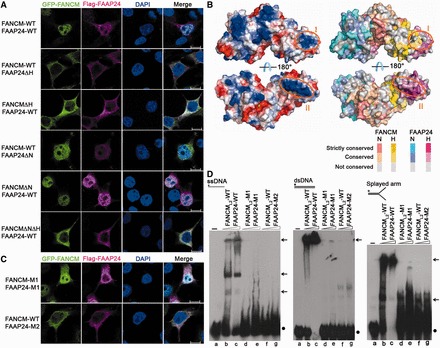
A potential DNA-binding site in FAAP24. (**A**) Localization of the wild-type and truncated FANCM-FAAP24. Scale bars: 10 µm. (**B**) Electrostatic surface (left panels) and distribution of the conserved residues on the surface (right panels) of FANCM-FAAP24. The two positively charged surface patches in the (HhH)_2_ domains are indicated with orange circles. The active site of FANCM is indicated by a modeled Mg^2+^ shown with a green sphere. The color coding scheme of the residue conservation is indicated. In the color coding scheme, the nuclease and the (HhH)_2_ domains are abbreviated as N and H, respectively. (**C**) Localization of the mutant FANCM-FAAP24. Scale bars: 10 µm. (**D**) EMSA of the wild-type and mutant FANCM-FAAP24. The reaction contained the biotin-labeled ssDNA, dsDNA and splayed-arm DNA (10 nM) without or with FANCM-FAAP24 (0.0 µM, lane a; 0.4 µM, lanes b, d and f; 0.8 µM, lanes c, e and g). The asterisks represent the biotin labels at the DNA 5′ end. The arrows indicate the shifted bands of the protein–DNA complexes, and the dots represent the free DNA probes.

The previous and our own biochemical data have shown that FANCM-FAAP24 could bind different types of DNA structures (5) (Figure 1A and Supplementary Figure S1). The previous biochemical data have shown that the (HhH)_2_ domain of FAAP24 is essential for targeting FANCM-FAAP24 to ICL DNA (24). Thus, we first investigated whether the (HhH)_2_ domains of FANCM and FAAP24 are essential for their localization to chromatin (Figure 4A). Our results show that the wild-type FANCM and FAAP24 co-localize to chromatin; however, deletion of the (HhH)_2_ domain of either FANCM (FANCM_1__–__1970_, FANCMΔH) or FAAP24 (FAAP24_1__–__153_, FAAP24ΔH) significantly reduces the localization to chromatin. In contrast, deletion of the nuclease domain of either FANCM (FANCM_Δ1813__–__1970_, FANCMΔN) or FAAP24 (FAAP24_153__–__215_, FAAP24ΔN) shows invisible or slight effect on the localization. Deletion of both nuclease and (HhH)_2_ domains of FANCM (FANCM_1__–__1813_, FANCMΔNΔH) disrupts the interactions with FAAP24 (Supplementary Figure S7B) and shows similar effect on chromatin localization as that of FANCMΔH. These results indicate that the (HhH)_2_ domains of FANCM and FAAP24 are critical for their binding to DNA and localization to chromatin.

We next tried to identify the potential DNA binding site(s) in the (HhH)_2_ domains of FANCM-FAAP24. Analysis of the electrostatic surface of the (HhH)_2_ domains reveals two positively charged surface patches (Figure 4B), and residues composing these patches are conserved in different vertebrate species (Supplementary Figure S4B and C). Region I is mainly composed of residues from helix α9 of FANCM and helix α9′ of FAAP24, both of which belong to the second HhH motif. Region II is mainly composed of residues from helix α5′ and the α5′-α6′ loop of the first HhH motif of FAAP24. Particularly, two basic residues (Lys171 and Lys173) in region II are highly conserved, and the strictly conserved Gly168-Val169-Gly170 residues form a GhG hairpin (where h indicates a hydrophobic residue) with their main-chain amino groups exposed on the surface (Supplementary Figure S7A), which may be equivalent to the GhG hairpin in ERCC1 and XPF that is involved in DNA binding (31,34,43).

To assess the functional importance of these two regions, we examined whether mutations in the two regions affect the localization of the FANCM-FAAP24 complex to chromatin and the binding ability to DNA (Figure 4C and D). The mutant complex containing mutations in region I (K1998A/R1999A of FANCM, FANCM-M1 and H208A of FAAP24, FAAP24-M1) shows slightly reduced localization to chromatin and an impaired DNA-binding activity, whereas the mutant complex containing mutations in region II (R161A/Q164A/K171A of FAAP24, FAAP24-M2) fails to localize to chromatin and abolishes the DNA-binding activity. The mutant complex containing the quadruple mutations R161A/Q164A/K171A/K173A of FAAP24 in region II exhibits similar effects as that containing the triple mutations (data not shown). We further confirmed that the mutations we introduced do not affect the proper folding of the proteins and the formation of the FANCM-FAAP24 complex both *in vivo* and *in vitro*. Our co-immunoprecipitation results show that the wild-type and mutant GFP-FANCM and Flag-FAAP24 form stable complexes (Supplementary Figure S7B), and our size-exclusion chromatography results show that the wild-type and mutant FANCM-FAAP24 complexes used in EMSA exist as heterodimers in solution (Supplementary Figure S7C).

The previous biological data have shown that FANCM can interact with the MHF1-MHF2 complex via the region following the helicase domain (residues 661–800) and with FAAP24 via the C-terminal region (residues 1799–2048) (3–5). To exclude the possible effect of the intrinsically expressed MHF1-MHF2 on the localization of FANCM to chromatin, we constructed a series of GFP-FANCM_L2_ in wild-type, truncated and mutant forms similar to those of full-length FANCM and examined the localization of FANCM-FAAP24 to chromatin. The immunofluorescence assay results show that the wild-type, truncated and mutant FANCM_L2_ exhibit similar chromatin localization patterns as the corresponding full-length FANCM, indicating that the interaction between the over-expressed GFP-FANCM and the intrinsically expressed MHF1-MHF2 has no notable effect on the localization of FANCM to chromatin (Supplementary Figure S7D). As both GFP-FANCM and Flag-FAAP24 were over-expressed in our assays and detected with the tag-specific antibodies, it is understandable that the signals for GFP-FANCM interacting with other intrinsically expressed protein partner(s) would be comparably very low and buried in the background. Our structural and functional data together indicate that region II (helix α5′ and the α5′-α6′ loop) of FAAP24 plays a critical role in DNA binding and is essential for the localization of FANCM-FAAP24 to chromatin. Most recently, Wienk *et al.* (48) reported the solution structure of the (HhH)_2_ domain of FAAP24 and showed that the first HhH motif is involved in the binding of ssDNA, which is consistent with our results. As region II of FAAP24 is independent of FANCM, we speculate that the DNA-binding ability of this region is not only essential for targeting FANCM-FAAP24 to chromatin but also for the independent function of FAAP24 in promoting ATR-mediated checkpoint activation (26).

The previous biochemical data demonstrated that FANCM-FAAP24 binds preferentially to ssDNA, splayed-arm DNA and a 3′-flap DNA substrate 5–10-fold better than to dsDNA (5), whereas FANCM-MHF binds preferentially to branched DNA and dsDNA (3,4). Both FAAP24 and MHF help to target FANCM to DNA. It is possible that FANCM-MHF-FAAP24 may work together as a molecular sensor, which scans along chromatin to detect stalled replication fork caused by ICLs (4). The two regions of FANCM interacting with MHF and FAAP24 may be located in proximity in the 3D structure such that MHF and FAAP24 could bind to dsDNA and ssDNA regions of one stalled replication fork, respectively, with high affinity and then cooperate to anchor FANCM to the branch point of the replication fork.

In summary, our structural and biological data demonstrate that like other XPF family members, both FANCM and FAAP24 consist of a nuclease domain and an (HhH)_2_ domain. The overall structure of FANCM-FAAP24 exhibits a similar architecture as that of ApXPF. Although the active site of FANCM has a similar structure as that of ApXPF and DrMus81, the variation of two key residues at the active site of FANCM and the inability of the surrounding region to bind DNA render a catalytically inactive nuclease domain. Moreover, our data indicate that helix α5′ and the α5′-α6′ loop of FAAP24 is a potential binding site for ssDNA and is critical for the localization of FANCM-FAAP24 to chromatin in DNA repair.

## ACCESSION NUMBERS

The coordinates and structure factors of the FANCM-FAAP24 complex have been deposited in the RCSB Protein Data Bank with the accession code 4M6W.

## SUPPLEMENTARY DATA

Supplementary Data are available at NAR Online.

## FUNDING

The Ministry of Science and Technology of China [2013CB910404 and 2011CB966301] and the National Natural Science Foundation of China [31230017 and 31221001]. Funding for open access charge: the Ministry of Science and Technology of China [2013CB910404].

*Conflict of interest statement*. None declared.

## Supplementary Material

Supplementary Data
