# Supplementary material for: Structural insights into the functions of the FANCM-FAAP24 complex in DNA repair
Source: Nucleic Acids Res. 2013 Sep 3;41(22):10573–83. doi: 10.1093/nar/gkt788 (PMC3905867; doi:10.1093/nar/gkt788)
Supplement: Supplementary Data [file supp_41_22_10573__index.html]

Structural insights into the functions of the FANCM-FAAP24 complex in DNA repair — Structural insights into the functions of the FANCM-FAAP24 complex in DNA repair — Supplementary Data 

# Structural insights into the functions of the FANCM-FAAP24 complex in DNA repair

## Supplementary Data

files

**Files in this Data Supplement:**

- Supplementary Data - pdf file
