# Supplementary material for: Structural insights into the functions of the FANCM-FAAP24 complex in DNA repair
Source: Nucleic Acids Res. 2013 Sep 3;41(22):10573–83. doi: 10.1093/nar/gkt788 (PMC3905867; doi:10.1093/nar/gkt788)
Supplement: Supplementary Data [file supp_gkt788_nar-01798-h-2013-File006.pdf]

Supplementary materials

Supplementary figure legends

Figure S1. Electrophoretic mobility shift assays of the FANCM-FAAP24 complex on (**A**) dsDNA and (**B**) splayed-arm DNA. The reaction contained the biotin-labeled DNA (10 nM) without or with increasing amounts of FANCM-FAAP24 (0.0 μM, lanes a, f, and k; 0.05 μM, lanes b, g, and l; 0.1 μM, lanes c, h, and m; 0.4 μM, lanes d, i, and n; and 0.8 μM, lanes e, j, and o). The asterisks represent the biotin labels at the DNA 5' end. The arrows indicate the shifted bands of the protein-DNA complexes and the dots represent the free DNA probes.

Figure S2. Structural comparison of FANCM and FAAP24. (**A**) Superposition of the nuclease domains of FANCM and FAAP24. (**B**) Superposition of the $(HhH)_2$ domains of FANCM and FAAP24. The secondary structure elements of FANCM are labeled. (**C**) Comparison of FANCM and FAAP24 based on superposition of the nuclease domains. The relative location and orientation of the nuclease and the $(HhH)_2$ domains in FANCM and FAAP24 are dramatically different. For reference, the nuclease and the $(HhH)_2$ domains of FANCM in the FANCM-FAAP24 complex are shown in gray cylinders. In the color coding scheme, the nuclease and the $(HhH)_2$ domains are abbreviated as N and H, respectively. The nuclease and the $(HhH)_2$ domains of FANCM are colored in salmon and yellow, and those of FAAP24 in cyan and violet, respectively.

Figure S3. Structural comparison of the FANCM-FAAP24 heterodimer, the ApXPF homodimer, and the DrMus81-HsEme1 heterodimer. The nuclease and the $(HhH)_2$ domains of FANCM are colored in salmon and yellow, and those of FAAP24 in cyan and violet, respectively. The ApXPF homodimer (PDF codes 2BGW and 2BHN) is colored with monomer A in light blue and monomer B in orange. The DrMus81-HsEme1 heterodimer

(PDB code 2ZIU) is colored with DrMus81 in magenta and HsEme1 in green. (**A**) Superposition of the individual nuclease domains. (**B**) Superposition of the individual $(HhH)_2$ domains. (**C**) Superimposition of the dimeric nuclease domains. (**D**) Superimposition of the dimeric $(HhH)_2$ domains. (**E**) Comparison of FANCM-FAAP24 and ApXFP-dsDNA (PDF code 2BGW). The bound dsDNA in ApXPF-dsDNA is shown with orange ribbon and the bound Mg^{2+} is shown with a green sphere to indicate the active site of the nuclease domain. ApXPF-dsDNA in the left panel is oriented by rotating 180º along the vertical axis to the plane in relation to that in the left panel of Figure 3B. Based on superposition of the two nuclease domains, the orientations of the two $(HhH)_2$ domains in the two complexes differ by a rotation of about 60º. (**F**) Comparison of the FANCM-FAAP24 heterodimer and the apo ApXPF homodimer (PDF code 2BHN). Based on superposition of the two nuclease domains, the orientations of the two $(HhH)_2$ domains in the two complexes differ by a rotation of about 125º. A modeled Mg^{2+} based on the ApXPF-dsDNA complex is shown with a green sphere to indicate the active site of the nuclease domain. (**G**) Comparison of the FANCM-FAAP24 heterodimer and the DrMus81-HsEme1 heterodimer. Based on superposition of the two nuclease domains, the orientations of the two $(HhH)_2$ domains in the two complexes also differ by a rotation of about 125º.

Figure S4. Sequence comparison of FANCM and FAAP24 from different species. (**A**) Structure-based sequence alignment of FANCM and FAAP24 with several members of the XPF family. Strictly conserved residues are highlighted with filled red boxes and conserved residues with open red boxes. The region corresponding to the GDX_nERKX_3D motif is highlighted with a blue box. (**B**) Sequence alignment of FAAP24 from different vertebrates. (**C**) Sequence alignment of FANCM from different vertebrates. The aligned sequences

include *Homo sapiens*, *Mus musculus*, *Gallus gallus*, *Xenopus laevis*, and *Danio rerio*. Residues composing the two positively charged surface patches on the $(HhH)_2$ domains of FANCM-FAAP24 are highlighted with green boxes.

Figure S5. Electrostatic surface representations of the active sites in HsXPF $(HhH)_2$-ssDNA (PDF code 2KN7) and DrMus81-HsEme1. HsXPF $(HhH)_2$-ssDNA is shown in the same orientation as in ApXPF-dsDNA in Figure 3B. HsXPF $(HhH)_2$-ssDNA in the lower panel and DrMus81-HsEme1 are shown in the same orientation as in Figure 3A. Mg^{2+} in these complexes was modeled based on ApXPF-dsDNA and is shown as a green sphere.

Figure S6. Electrophoretic mobility shift assays of the wild-type and dimeric nuclease domains of FANCM-FAAP24. The reaction contained the biotin-labeled ssDNA, dsDNA, and splayed-arm DNA (10 nM) without or with FANCM-FAAP24 (0.0 μM, lanes a and e; 0.1 μM, lanes b and f; 0.4 μM, lanes c and g; 0.8 μM, lanes d and h). The asterisks represent the biotin labels at the DNA 5' end. The arrows indicate the shifted bands of the protein-DNA complexes and the dots represent the free DNA probes.

Figure S7. Identification of the potential DNA-binding site in FANCM-FAAP24. (A) Electrostatic surface of the potential DNA-binding site in the $(HhH)_2$ domain of FAAP24. Basic and acidic surfaces are colored in blue and red, respectively. Residues that may participate in DNA binding are shown with ball-and-stick models. (B) Flag pull-down assays were performed on lysates of HEK 293T cells co-expressing GFP-FANCM and Flag-FAAP24 in wild-type, truncated, and mutant forms. Soluble fractions of each cell lysate were incubated with anti-Flag M2 affinity gel (Sigma). Input and pull-down fraction were analyzed by western blot and probed with indicated antibodies. Mouse anti-flag M2 mAb (1:1000, Sigma) and rabbit anti-GFP antibody (1:1000, TIANGEN) along with horseradish

peroxidase conjugated anti-mouse antibody (1:3000, Invitrogen) or anti-rabbit antibody (1:3000, Promega) were used to detect Flag-FAAP24 and GFP-FANCM, respectively. (C) Analyses of the oligomeric states of the wild-type and mutant FANCM-FAAP24 complexes in solution by size-exclusion chromatography. The positions of the protein markers are labeled. The curves for the wild-type FANCM-FAAP24, FANCM-M1-FAAP24-M1 and FANCM-FAAP24-M2 are colored in blue, red, and green, respectively. (D) Localization of the C-terminal segment of FANCM ($FANCM_{L2}$) and FAAP24 in wild-type, truncated and mutant forms. Scale bars: 10 μm.

Table S1. Interactions at the interface of the nuclease and the $(HhH)_2$ domains of FANCM

(A) Hydrogen bonds (≤ 3.6 Å)

Nuclease	Location	Distance (Å)	$(HhH)_2$	Location
Asn1859-$O^{\delta 1}$	β3/β4 loop	3.2	Asn1989-$N^{\delta 2}$	α8
Glu1892-$O^{\varepsilon 2}$	α4/β5 loop	3.6	Thr2015-N	α10/α11 loop

(B) van der Waals contacts (≤ 4.0 Å)

Nuclease	Location	$(HhH)_2$	Location
Leu1821 (1) [a]	β1	Tyr1984 (1)	α8
Cys1848 (2)	β2	Tyr1984 (2)	α8
Pro1849 (2)	β2/β3 loop	Tyr1984 (2)	α8
Leu1850 (3)	β2/β3 loop	Tyr1984 (3)	α8
Ile1856 (1)	β3	Ile1985 (1)	α8
Ser1858 (1)	β3/β4 loop	Ile1985 (1)	α8
Asn1859 (8)	β3/β4 loop	Ile1985 (2)	α8
		Asn1989 (2)	α8
		Gln2013 (4)	α10/α11 loop
Ser1889 (7)	α4	Asn1981 (5)	α7/α8 loop
		Lys2018 (2)	α11
Met1890 (6)	α4	Asn1981 (1)	α7/α8 loop
		Ile1982 (3)	α7/α8 loop
		Ser1983 (2)	α7/α8 loop
Phe1891 (3)	α4/β5 loop	Ser1983 (3)	α7/α8 loop
Glu1892 (4)	α4/β5 loop	Val2014 (1)	α10/α11 loop
		Thr2015 (3)	α10/α11 loop
Ile1959 (3)	α6/α7 loop	Ile1985 (3)	α8
Pro1962 (7)	α6/α7 loop	His1992 (7)	α8/α9 loop
Thr1963 (2)	α6/α7 loop	Leu1988 (2)	α8

[a] There are a total of 50 van der Waals contacts. Numbers in parentheses refer to the number of van der Waals contacts involved.

Table S2. Interactions at the interface of FANCM and FAAP24

(A) Hydrogen bonds and salt bridge (≤ 3.6 Å)

FANCM	Location	Distance (Å)	FAAP24	Location
**Nuclease domain**				
Gln1885-$N^{\varepsilon 2}$	α4	2.9	Pro144-O	α4'/α5' loop
Gln1888-$N^{\varepsilon 2}$	α4	3.1	Leu145-O	α4'/α5' loop
Lys1900-N^{η}	β5/α5 loop	3.6	Glu98-$O^{\varepsilon 2}$	α3'
Arg1931-$N^{\eta 2}$	β6	3.5	Gly113-O	α3'/β6' loop
Phe1934-N	β6	2.8	Gln105-$O^{\varepsilon 1}$	α3'
Phe1934-O	β6	2.9	Gln105-$N^{\varepsilon 2}$	α3'
**$(HhH)_2$ domain**				
Ser1978-O^{γ}	α7	2.7	Thr162-$O^{\gamma 1}$	α5'
Ser1996-O^{γ}	α9	3.2	Gln213-N	C-terminal
Lys1998-N	α9	3.4	Phe211-O	α9'
Asn2002-$O^{\delta 1}$	α9	3.5	Gln185-$N^{\varepsilon 2}$	α7'
Asn2002-$O^{\delta 1}$	α9	3.0	Asn189-$N^{\delta 2}$	α7'/α8' loop
Asn2002-$N^{\delta 2}$	α9	3.4	Ser188-O^{γ}	α7'
Tyr2025-O^{η}	α11	2.7	Thr162-$O^{\gamma 1}$	α5'
Ile2026-O	α11	3.3	Ile184-N	α7'
Ile2026-O	α11	3.2	Gln185-N	α7'
Tyr2028-O^{η}	C-terminal	2.9	Glu156-$O^{\varepsilon 2}$	α5'
Phe2030-N	C-terminal	3.3	Pro182-O	α6'/α7' loop

(B) van der Waals contacts (≤ 4.0 Å)

FANCM	Location	FAAP24	Location
**Nuclease domain**			
Ile1881 (3) [a]	α4	Pro144 (1)	α4'/α5' loop
		Leu145 (2)	α4'/α5' loop
Ile1884 (2)	α4	Leu145 (2)	α4'/α5' loop
Gln1885 (3)	α4	Pro144 (2)	α4'/α5' loop
		Leu145 (1)	α4'/α5' loop
Gln1888 (6)	α4	Leu145 (6)	α4'/α5' loop
Lys1900 (3)	β5/α5 loop	Lys93 (3)	β5'/α3' loop
Asp1919 (2)	α5	Leu128 (2)	α4'
Ser1920 (1)	α5	Gln131 (1)	α4'
Thr1923 (7)	α5	Gln131 (1)	α4'

		Leu132 (2)	α4’
		Glu135 (4)	α4’
Thr1924 (4)	α5	Asn143 (2)	α4’/α5’ loop
		Leu145 (2)	α4’/α5’ loop
Ile1926 (3)	α5	Val115 (2)	β6’
		Leu117 (1)	β6’
Gly1927 (1)	α5	Leu146 (1)	α4’/α5’ loop
Ala1928 (2)	α5	Leu145 (2)	α4’/α5’ loop
Ile1930 (1)	α5/β6 loop	Leu145 (1)	α4’/α5’ loop
Leu1933 (9)	β6	Gln105 (6)	α3’
		Val109 (2)	α3’
		Leu110 (1)	α3’
Phe1934 (16)	β6	Gln105 (8)	α3’
		Leu116 (1)	β6’
		Pro118 (7)	β6’
Glu1947 (5)	α6	Lys106 (1)	α3’
		Leu110 (4)	α3’
Leu1948 (2)	α6	Leu110 (2)	α3’
**(HhH)$_2$ domain**			
Gln1974 (3)	α7	Gln165 (3)	α5’
Phe1975 (12)	α7	Thr162 (5)	α5’
		Gln165 (2)	α5’
		Ile166 (2)	α5’/α6’ loop
		Phe210 (3)	α9’
Ser1978 (7)	α7	Thr162 (7)	α5’
Ile1979 (1)	α7/α8 loop	Thr162 (1)	α5’
Pro1980 (2)	α7/α8 loop	Ser158 (2)	α5’
Ser1995 (2)	α8/α9 loop	Gln213 (2)	C-terminal
Ser1996 (7)	α8/α9 loop	Phe211 (2)	C-terminal
		Gln213 (5)	C-terminal
Val1997 (4)	α9	Phe210 (2)	α9’
		Phe211 (2)	C-terminal
Lys1998 (9)	α9	Ser188 (1)	α7’
		Asn189 (2)	α7’/α8’ loop
		Phe211 (6)	C-terminal
Ala2001 (2)	α9	Ser188 (2)	α7’
Asn2002 (3)	α9	Gln185 (1)	α7’
		Asn189 (2)	α7’/α8’ loop
Arg2024 (2)	α11	Leu154 (2)	α4’/α5’ loop
Tyr2025 (15)	α11	Leu154 (1)	α4’/α5’ loop

		Ser158 (3)	α5’
		Leu159 (8)	α5’
		Thr162 (3)	α5’
Ile2026 (6)	α11	Ser183 (1)	α6’/α7’ loop
		Ile184 (3)	α7’
		Gln185 (2)	α7’
His2027 (7)	α11	Gln185 (7)	α7’
Tyr2028 (10)	C-terminal	Leu154 (1)	α4’/α5’ loop
		Glu156 (4)	α5’
		Leu159 (1)	α5’
		Pro182 (2)	α6’/α7’ loop
		Ser183 (2)	α6’/α7’ loop
Val2029 (3)	C-terminal	Pro182 (2)	α6’/α7’ loop
		Gln186 (1)	α7’
Phe2030 (14)	C-terminal	Glu156 (9)	α5’
		Leu178 (2)	α6’
		Pro182 (3)	α6’/α7’ loop

[a] There are a total of 179 van der Waals contacts. Numbers in parentheses refer to the number of van der Waals contacts involved.

Figure S1

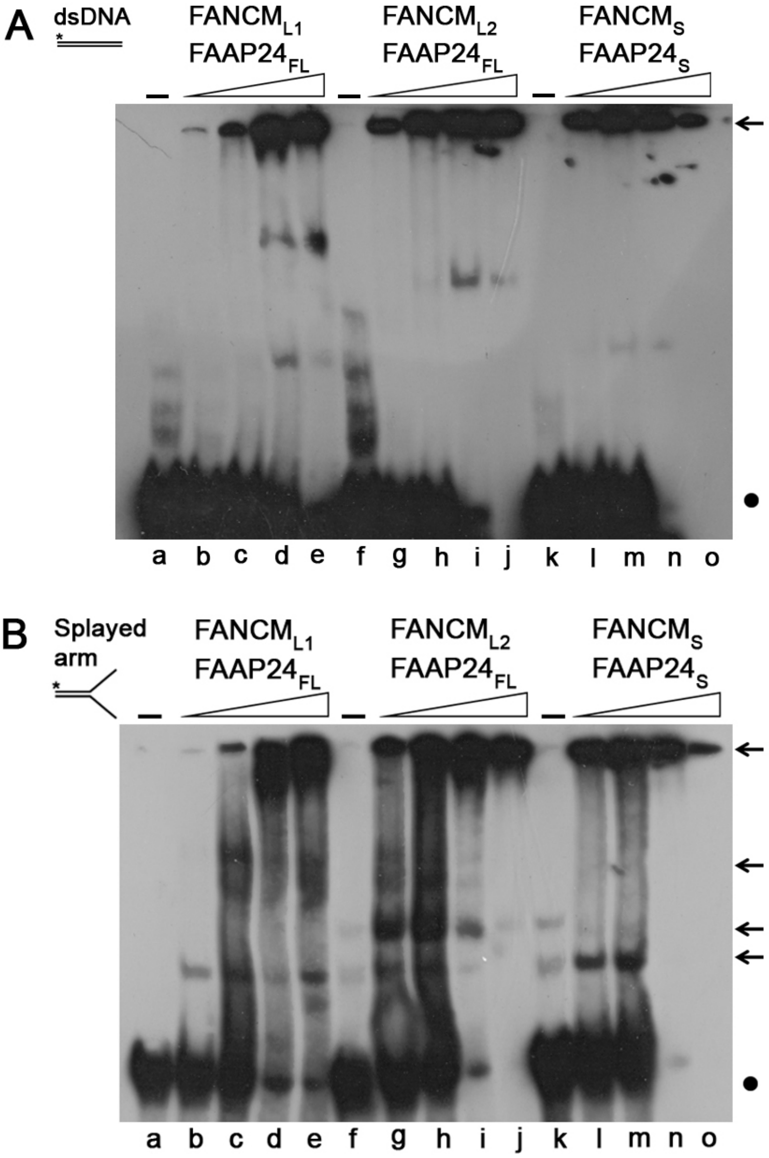
A
dsDNA
FANCM$_{L1}$
FAAP24$_{FL}$
FANCM$_{L2}$
FAAP24$_{FL}$
FANCM$_{S}$
FAAP24$_{S}$
a b c d e f g h i j k l m n o
B
Splayed arm
FANCM$_{L1}$
FAAP24$_{FL}$
FANCM$_{L2}$
FAAP24$_{FL}$
FANCM$_{S}$
FAAP24$_{S}$
a b c d e f g h i j k l m n o

Figure S2

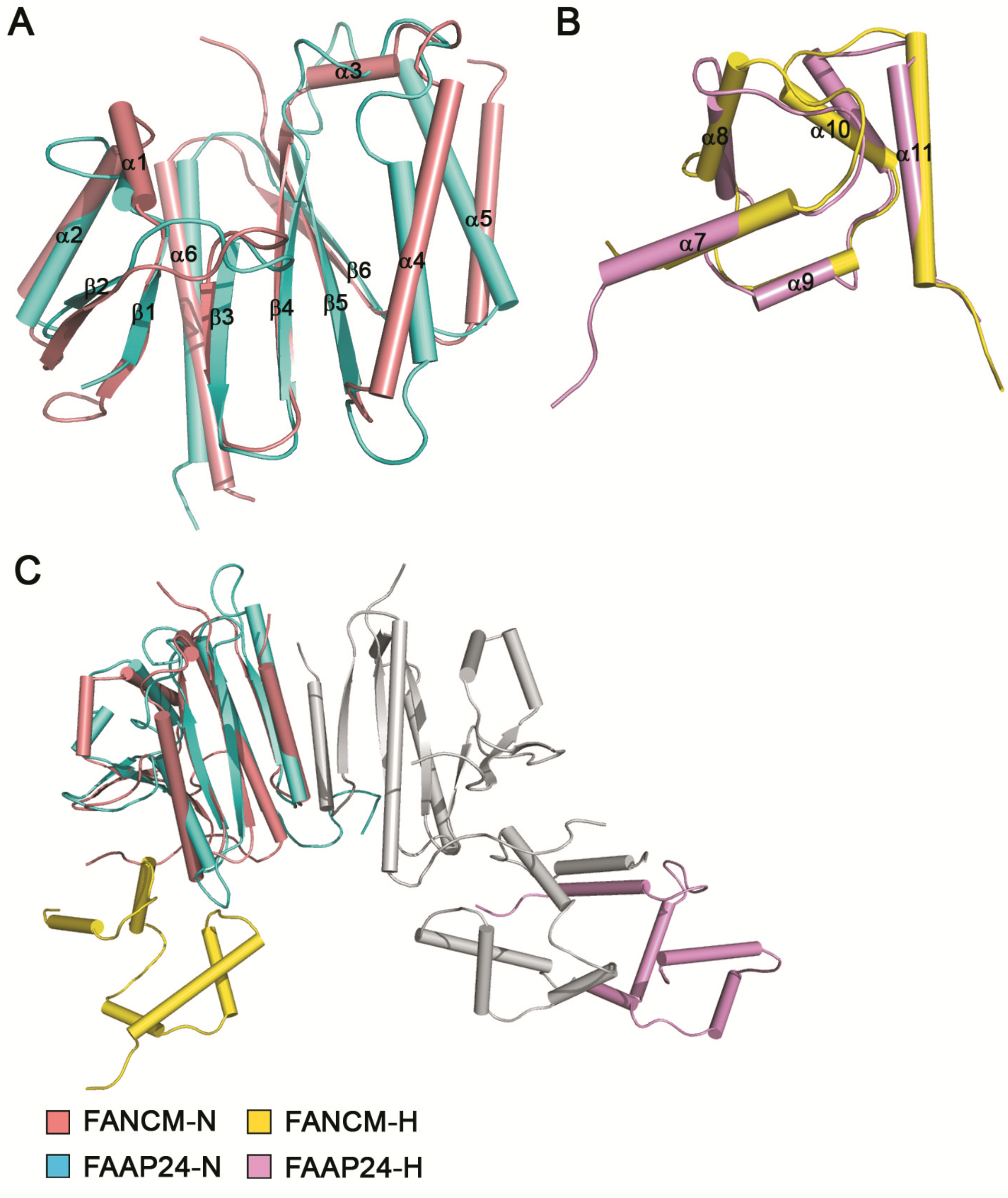
A
B
C
α1
α2
α3
α4
α5
α6
β1
β2
β3
β4
β5
β6
α7
α8
α9
α10
α11
FANCM-N
FANCM-H
FAAP24-N
FAAP24-H

Figure S3

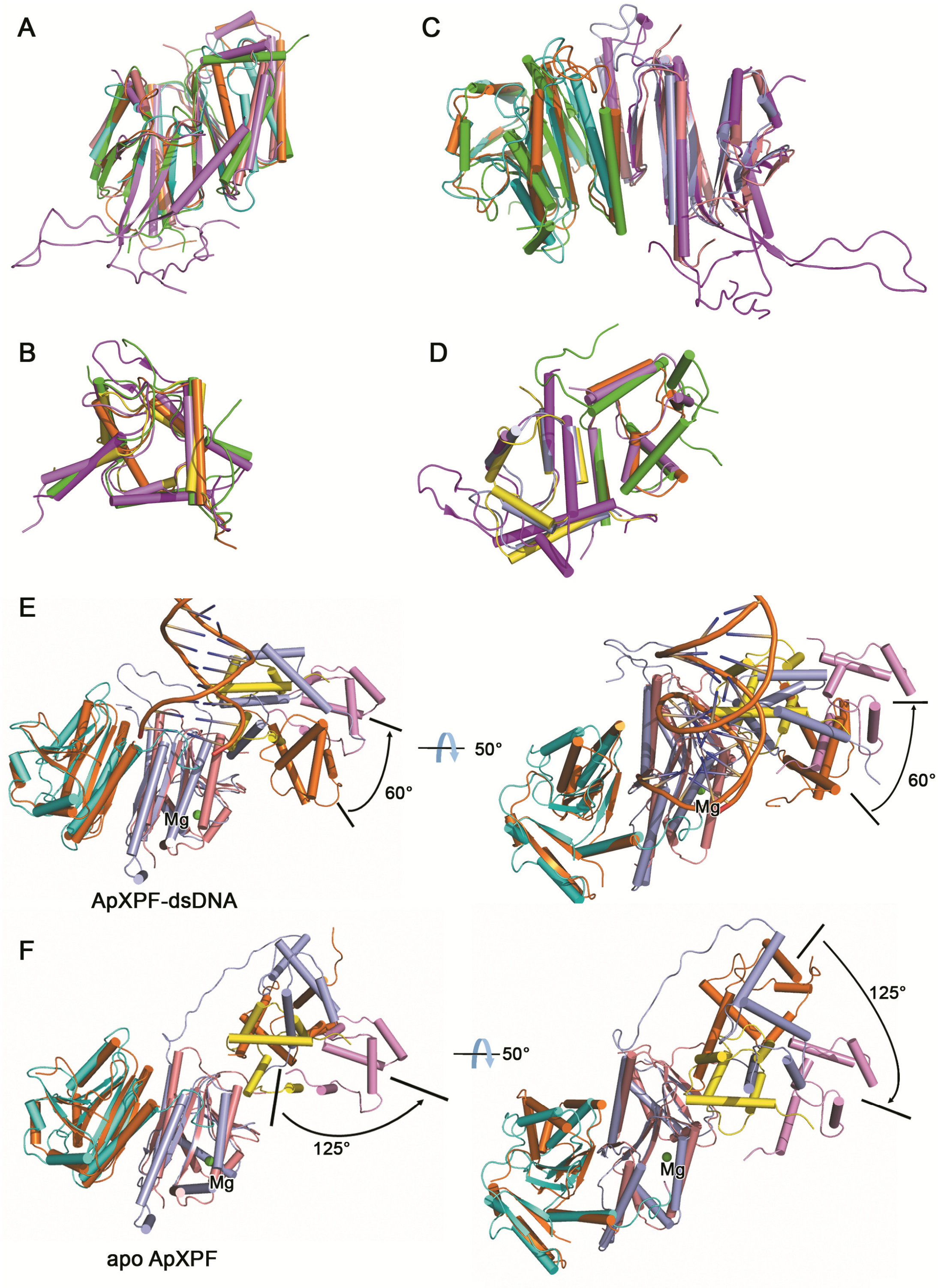

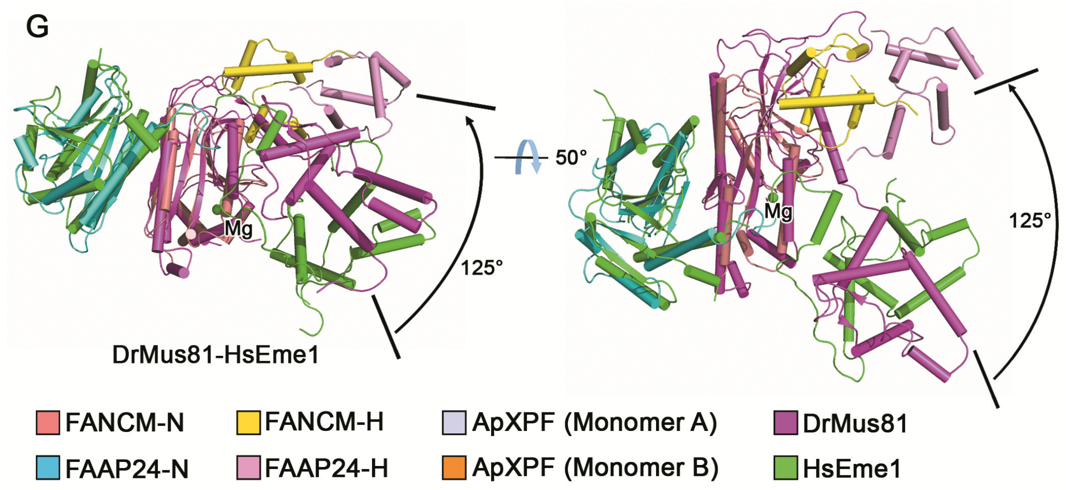
G
Mg
125°
50°
DrMus81-HsEme1
Mg
125°
FANCM-N
FANCM-H
ApXPF (Monomer A)
DrMus81
FAAP24-N
FAAP24-H
ApXPF (Monomer B)
HsEme1

Figure S4

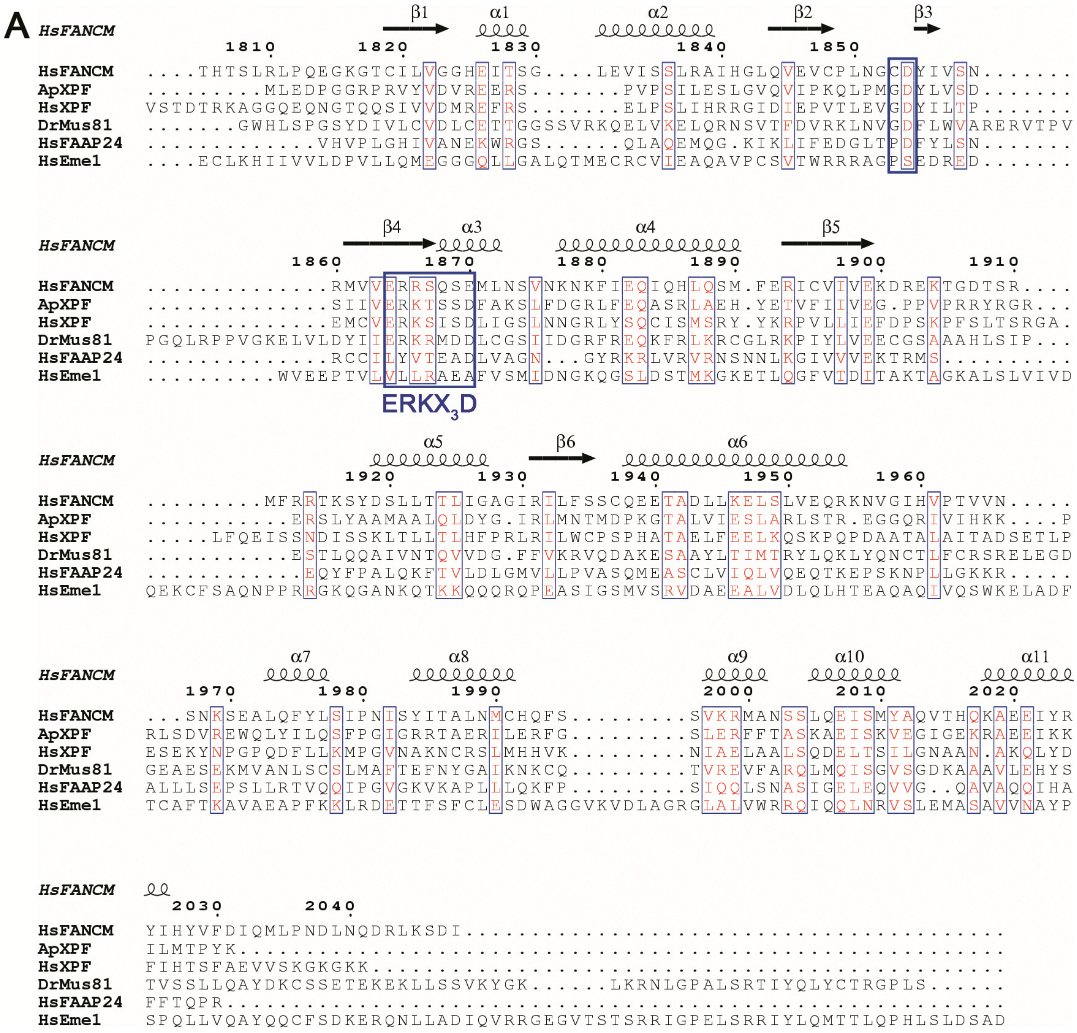

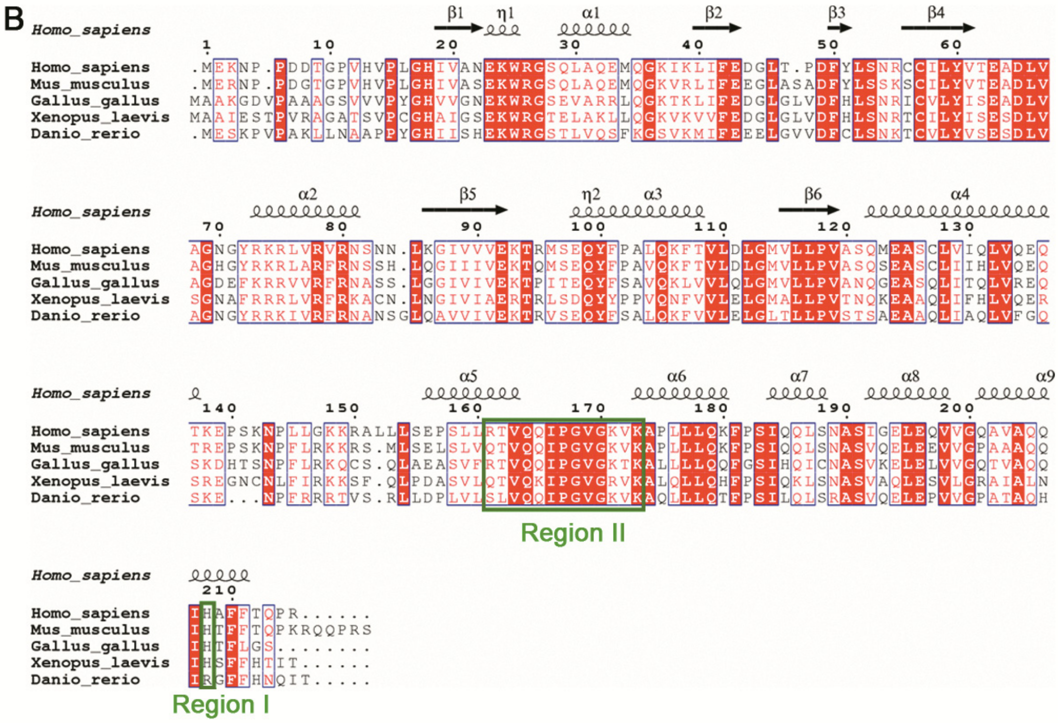
B
Homo_sapiens
Mus_musculus
Gallus_gallus
Xenopus_laevis
Danio_rerio
Region II
Region I

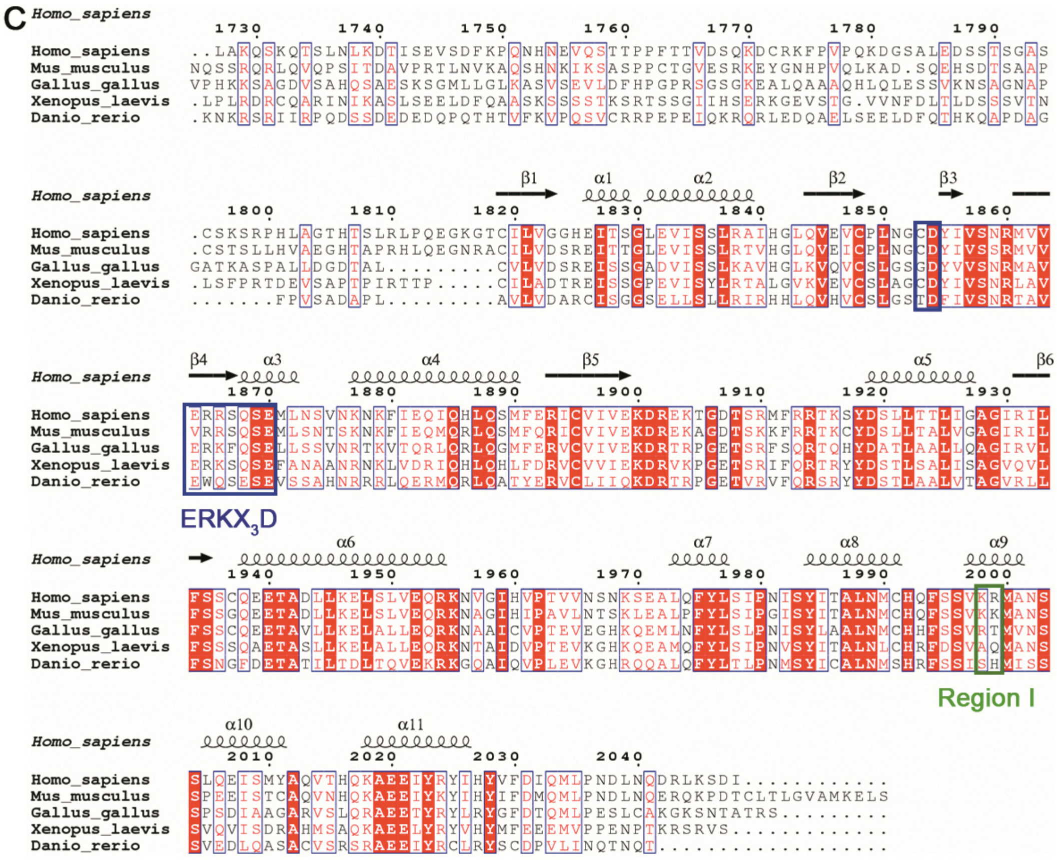
C
Homo_sapiens
Mus_musculus
Gallus_gallus
Xenopus_laevis
Danio_rerio
ERKX3D
Region I

Figure S5

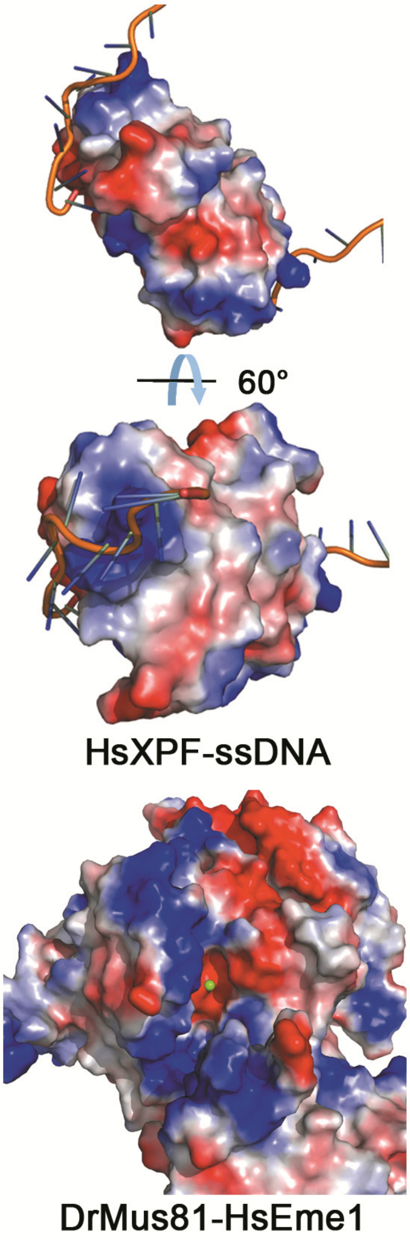

Figure S6

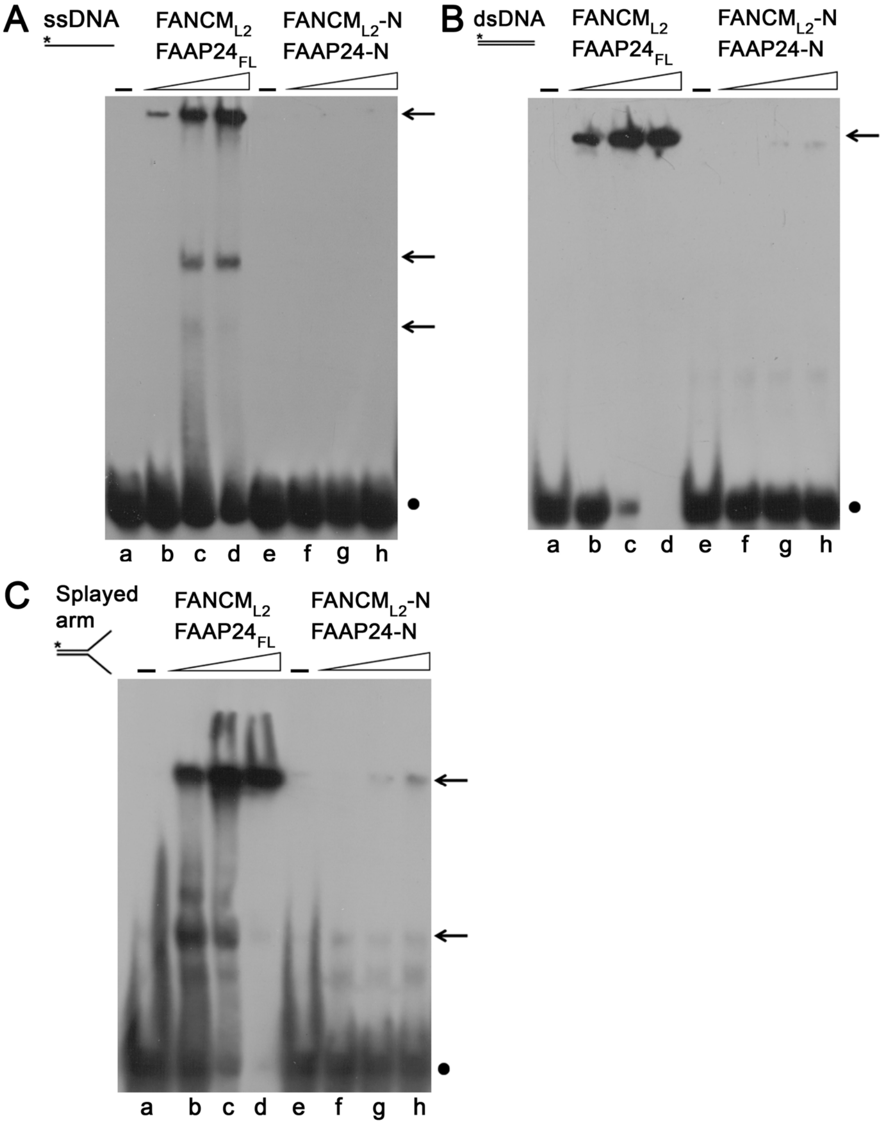
A
ssDNA
FANCM$_{L2}$
FAAP24$_{FL}$
FANCM$_{L2}$-N
FAAP24-N
a b c d e f g h
B
dsDNA
FANCM$_{L2}$
FAAP24$_{FL}$
FANCM$_{L2}$-N
FAAP24-N
a b c d e f g h
C
Splayed arm
FANCM$_{L2}$
FAAP24$_{FL}$
FANCM$_{L2}$-N
FAAP24-N
a b c d e f g h

Figure S7

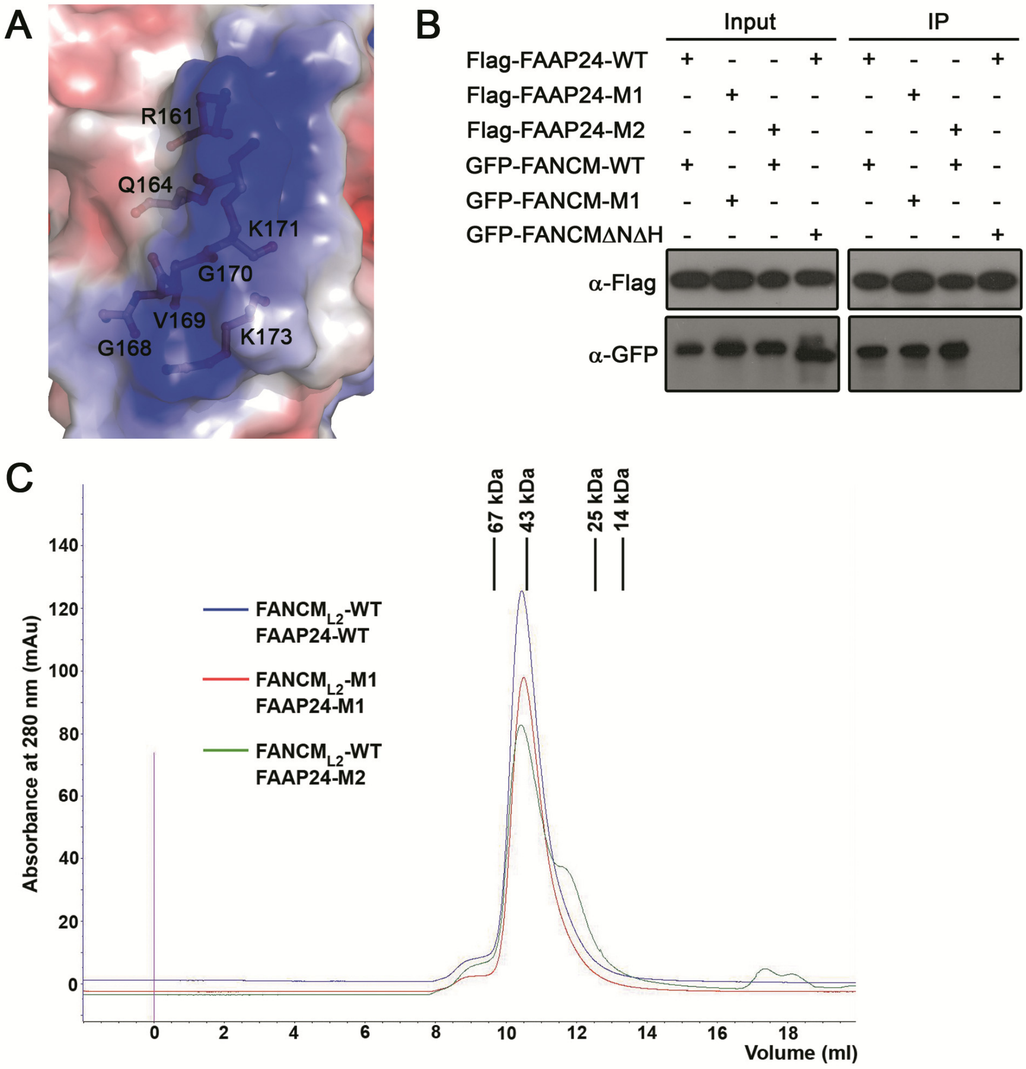
A
R161
Q164
K171
G170
V169
K173
G168
B
Input
IP
Flag-FAAP24-WT + - - + + - - +
Flag-FAAP24-M1 - + - - - + - -
Flag-FAAP24-M2 - - + - - - + -
GFP-FANCM-WT + - + - + - + -
GFP-FANCM-M1 - + - - - + - -
GFP-FANCMΔNΔH - - - + - - - +
α-Flag
α-GFP
C
67 kDa
43 kDa
25 kDa
14 kDa
Absorbance at 280 nm (mAu)
FANCM$_{L2}$-WT FAAP24-WT
FANCM$_{L2}$-M1 FAAP24-M1
FANCM$_{L2}$-WT FAAP24-M2
Volume (ml)

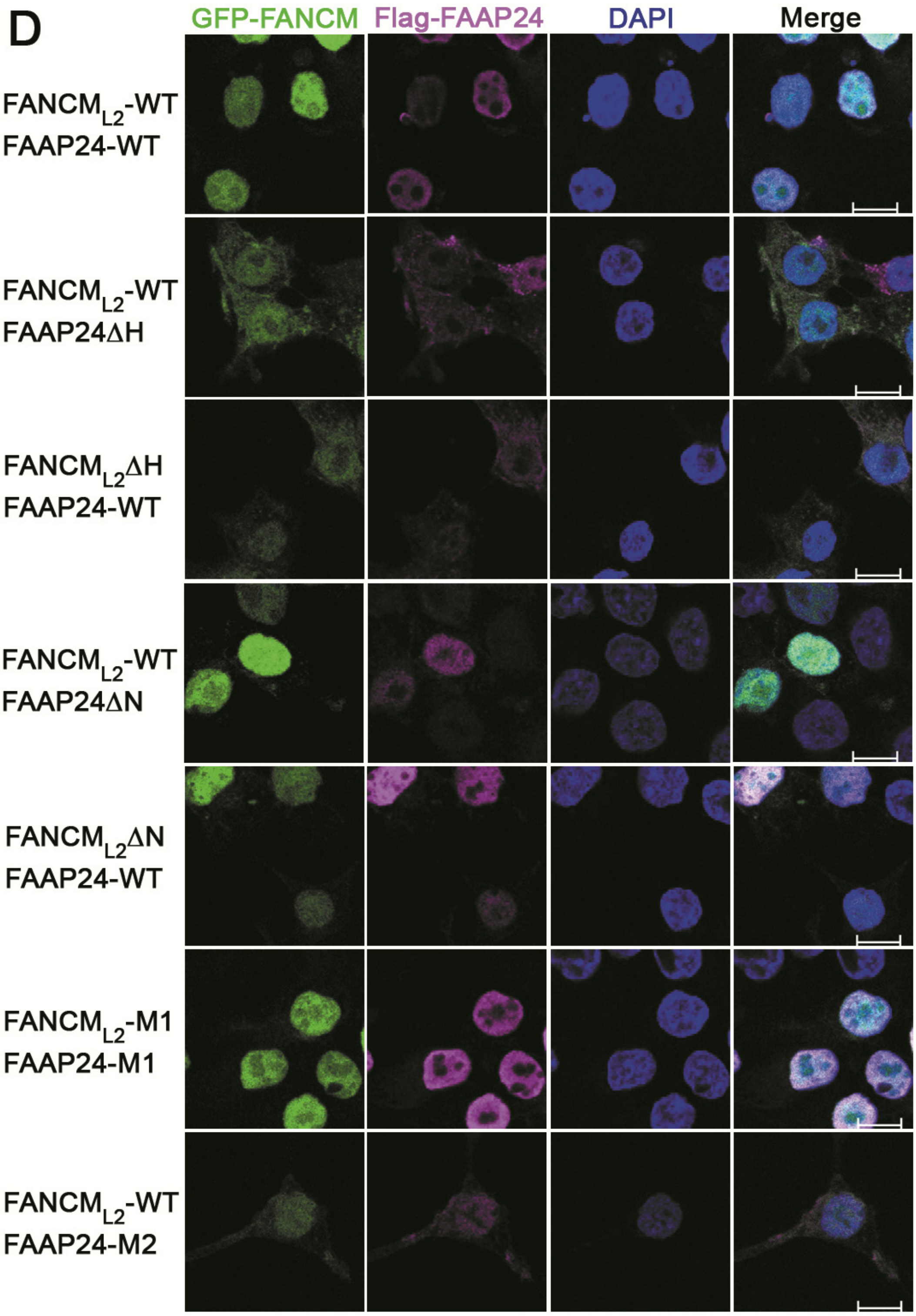
D
GFP-FANCM
Flag-FAAP24
DAPI
Merge
FANCML2-WT
FAAP24-WT
FANCML2-WT
FAAP24ΔH
FANCML2ΔH
FAAP24-WT
FANCML2-WT
FAAP24ΔN
FANCML2ΔN
FAAP24-WT
FANCML2-M1
FAAP24-M1
FANCML2-WT
FAAP24-M2
